# Supplementary material for: Mapping the evolution of fertility support policies in China: A content and instrumental analysis
Source: PLoS One. 2025 Oct 9;20(10):e0332137. doi: 10.1371/journal.pone.0332137 (PMC12510515; doi:10.1371/journal.pone.0332137)
Supplement: S1 Appendix — (ZIP) [file pone.0332137.s001.zip › S1 Appendix. 226 original policy documents/221-财政部、卫生部关于印发《农村孕产妇住院分娩专项补助资金管理暂行办法》的通知(FBM-CLI-4-122195).doc]

财政部、卫生部关于印发《农村孕产妇住院分娩专项补助资金管理暂行办法》的通知

**发布部门：** [财政部](https://www.pkulaw.com/law/chl?Aggs.IssueDepartment=60210) [卫生部(已撤销)](https://www.pkulaw.com/law/chl?Aggs.IssueDepartment=60222) [机构沿革](javascript:void(0))

**发文字号：**财社〔2009〕36号

**发布日期：**2009.06.01

**实施日期：**2009.06.01

**时效性：** [失效](https://www.pkulaw.com/law/chl?Aggs.TimelinessDic=02)

**效力级别：** [部门规范性文件](https://www.pkulaw.com/law/chl?Aggs.EffectivenessDic=XE0302)

**法规类别：** [专项资金管理](https://www.pkulaw.com/law/chl?Aggs.Category=03116) [妇幼卫生](https://www.pkulaw.com/law/chl?Aggs.Category=09622)

**失效依据：**[公共卫生服务补助资金管理暂行办法](javascript:TMP(269175,0,'chl'))

财政部、卫生部关于印发《农村孕产妇住院分娩专项补助资金管理暂行办法》的通知
（2009年6月1日　财社[2009]36号）

各省、自治区、直辖市、计划单列市财政厅（局）、卫生厅（局）：
　　为规范和加强农村孕产妇住院分娩专项补助资金管理，我们制定了《农村孕产妇住院分娩专项补助资金管理暂行办法》，现印发给你们，请遵照执行。执行中发现问题，请及时反馈财政部、卫生部。
　　附件：农村孕产妇住院分娩专项补助资金管理暂行办法
　　附件：

　　农村孕产妇住院分娩专项补助资金管理暂行办法

　第一章　总则

　　**第一条**　为规范农村孕产妇住院分娩专项补助资金（以下简称专项补助资金）的管理，提高资金使用效益，制定本暂行办法。

　第二章　资金筹集

　　**第二条**　本办法所称专项补助资金是指各级财政根据卫生部、[财政部《关于进一步加强农村孕产妇住院分娩工作的指导意见](https://www.pkulaw.com/chl/f4a349efdd46402cbdfb.html)》（卫妇社发[2009]12号）有关规定安排的专项用于农村孕产妇住院分娩的补助资金。

　　**第三条**　财政部门要做好农村孕产妇住院分娩补助资金的预算安排。县级财政应将本地区农村孕产妇住院分娩补助所需资金纳入医疗卫生经费预算予以安排。省级财政要安排必要的专项补助资金，支持困难地区落实农村孕产妇住院分娩补助政策。

　　**第四条**　中央财政安排专项补助资金，对困难地区予以支持。中央财政专项补助资金，按照“当年金额预拨、次年考核结算、差额多退少补”原则下达。年初按上年度农村孕产妇住院分娩人数和当年确定的补助标准预拨当年补助资金，次年按各地上报的截至上年末实际住院分娩农村孕产妇人数，并统筹考虑各地农村孕产妇住院分娩工作的绩效考核情况，结算上年度中央财政专项补助资金。多拨资金抵顶当年预拨资金，少拨资金予以补足。绩效考核以国家规定的分省工作目标为基础，重点考核农村孕产妇实际住院分娩人数、住院分娩率、孕产妇死亡率和婴儿死亡率等工作指标。

　第三章　资金使用

　　**第五条**　农村孕产妇住院分娩补助对象必须具备以下条件：
　　（一）农业户籍；
　　（二）在定点医疗卫生机构住院分娩；
　　（三）符合国家有关政策。

　　**第六条**　省级卫生、财政部门根据卫生部、财政部制定的农村孕产妇住院分娩基本服务项目，确定本地区的服务项目和限价标准，并结合本地经济社会发展水平和财政承受能力，合理确定本地区农村孕产妇住院分娩的人均财政补助标准。享受中央财政补助地区确定的人均补助标准不得低于中央财政人均补助标准。

　　**第七条**　县级卫生部门会同财政部门确定当地具备助产资质条件的定点医疗卫生机构（包括社会力量举办的医疗卫生机构），承担农村孕产妇住院分娩任务。定点医疗卫生机构名单要通过适当方式向社会公开，以方便农村孕产妇选择适合自己的定点医疗卫生机构。

　　**第八条**　定点医疗卫生机构对住院分娩的农村孕产妇，按人均财政补助标准免收住院分娩的相关费用。定点医疗卫生机构定期向县级卫生部门报送农村孕产妇住院分娩人数等情况，经县级卫生、财政部门审核批准后，由财政部门将专项补助资金通过国库集中支付方式直接拨付给定点医疗卫生机构。也可委托中介机构进行审核，经卫生、财政部门核准后拨付。异地住院分娩的农村孕产妇补助办法由各地根据实际情况另行制定。

　　**第九条**　定点医疗卫生机构应严格按规定执行基本服务项目和收费标准，如收取规定服务项目之外的其他服务费用，需征得孕产妇本人或家属同意。参加新型农村合作医疗的农村孕产妇在财政补助标准之外的住院分娩费用，可按当地新型农村合作医疗制度的相关规定给予补偿。对个人负担较重的贫困孕产妇，可按规定由农村医疗救助制度给予救助。

　　**第十条**　有条件的地区，可探索将农村孕产妇住院分娩专项补助资金与新型农村合作医疗基金统筹管理，由新型农村合作医疗经办机构根据审核结果，将补助资金支付给定点医疗卫生机构等有效途径。

　第四章　监督管理

　　**第十一条**　县级卫生部门负责农村孕产妇住院分娩的日常管理工作；负责定点医疗卫生机构和人员服务资质管理；开展相关宣传和健康教育工作，引导农村孕产妇住院分娩；审核农村孕产妇住院分娩的相关材料，做好农村孕产妇住院分娩的基础信息统计、分析和管理工作。

　　**第十二条**　县级卫生部门要会同财政部门加强对定点医疗卫生机构产科服务质量、农村孕产妇住院分娩专项补助资金使用等情况的绩效考评，有条件的地区，可委托有资质的中介机构对定点医疗卫生机构实施绩效考评。对经考评达不到规范要求的医疗卫生机构，要按有关规定取消其定点资格。同时，要将考评结果与专项补助资金挂钩。

　　**第十三条**　专项补助资金必须专项用于农村孕产妇住院分娩补助，任何单位和个人不得以任何形式截留、挤占和挪用专项补助资金。农村孕产妇住院分娩专项补助资金的管理使用，以及享受补助的孕产妇等情况应定期向社会公布，接受群众监督。对故意虚报有关数字和情况骗取专项补助资金，或截留、挤占和挪用专项补助资金的，要按照《[财政违法行为处罚处分条例](https://www.pkulaw.com/chl/8c9f093bbfbeef99bdfb.html)》（国务院令第427号）等有关法律法规，除责令改正、追回有关财政资金外，还要按规定追究有关单位和人员责任。

　　**第十四条**　省级财政、卫生部门要及时将中央财政专项补助资金分配使用情况上报财政部、卫生部，有关资金分配文件要同时抄送财政部驻当地财政监察专员办事处。

　第五章　附则

　　**第十五条**　省级财政、卫生部门可结合本地实际，制定本地区专项补助资金的拨付和管理办法。

　　**第十六条**　本暂行办法自印发之日起执行，由财政部会同卫生部负责解释。

© 北大法宝：（[www.pkulaw.com](https://www.pkulaw.com/)) 专业提供法律信息、法学知识和法律软件领域各类解决方案。北大法宝为您提供丰富的参考资料，正式引用法规条文时请与标准文本核对。
欢迎查看所有[产品和服务](https://www.pkulaw.com/helps/2.html)。法宝快讯：[如何快速找到您需要的检索结果？](https://www.pkulaw.com/helps/70.html)[法宝](https://www.pkulaw.com/helps/69.html)V6有何新特色？

扫描二维码阅读原文

**原文链接：**<https://www.pkulaw.com/chl/faac9c20b53916f1bdfb.html>
